# Quality in dementia care: A cross sectional study on the Bio-Psycho-Social competencies of health care professionals

**DOI:** 10.1371/journal.pone.0191440

**Published:** 2018-02-01

**Authors:** Patricia De Vriendt, Elise Cornelis, Valerie Desmet, Ruben Vanbosseghem, Dominique Van de Velde

**Affiliations:** 1 Department of Occupational Therapy and Department Nursing, Research group Innovation in Health Care, Artevelde University College, Ghent, Belgium; 2 Faculty of Medicine and Health Sciences, Department of Rehabilitation Sciences and Physiotherapy, Occupational therapy program, Ghent University, Ghent; 3 Department Gerontology and Frailty in Ageing (FRIA) Research Group Vrije Universiteit Brussel, Belgium; 4 University Hospital Brussel, Belgium; Istituto Di Ricerche Farmacologiche Mario Negri, ITALY

## Abstract

**Objective:**

Professionals in dementia-care ought to be able to work within a Bio-Psycho-Social model. The objectives were to examine whether dementia-care is delivered in a Bio-Psycho-Social way, to explore the influencing factors and to evaluate the factorial validity of the ‘Bio-Psycho-Social-Dementia-Care scale’.

**Design and setting:**

413 healthcare-professionals completed the ‘Bio-Psycho-Social-Dementia-Care scale’. Differences between groups (settings, professions, years of experience) were calculated with a student’s t-test and one-way ANOVA. The facture structure of the scale was evaluated using a confirmatory factor analysis.

**Results:**

The factor-analysis confirmed the 5 subscale-structure (1) networking, (2) using the client’s expertise, (3) assessment and reporting, (4) professional knowledge and skills and (5) using the environment. (No significant differences were found between professionals in residential care and community care for the subscales ‘networking’ and ‘using the client’s expertise’. Professionals in residential care score higher than community care for ‘assessment and reporting’ (p<0,05) and ‘professional knowledge and skills’ (p<0,01) but lower for ‘using the environment’ (p<0,001). The juniors score higher for ‘professional knowledge’ compared to seniors (p<0,01) and the seniors score better for ‘professional experience’ (p<0,01). The Cure and Care disciplines and the Therapy disciplines had higher values in ‘assessment and reporting’ compared to the Social Support disciplines (p<0,001 and p<0.001). The Therapy disciplines scored higher in ‘using professional knowledge and skills’ compared to the Social Support group (p 0.021) and the Cure and Care disciplines (p<0,001). The Social Support disciplines scored higher in ‘using the environment’ compared to the Therapy disciplines (p<0.001) and the Cure and care disciplines (p<0.001).

**Conclusion:**

The Bio-Psycho-Social-Dementia-scale is a valid tool and offers opportunities not only to rate, but also to improve Bio-Psycho-Social functioning in dementia-care: increase interdisciplinary collaboration, facilitate assessment, combine the strengths of the different professions and install a heterogeneous team with regard to age and experience.

## Introduction

Healthcare provision is changing from a primarily biomedical towards a more bio-psycho-social conception of disability and health [1–4]. A bio-psycho-social model is originally defined as a model including both the person and the illness in the reasoning process of the healthcare professional [5]. This model focusses on social participation and daily activity performance despite illness and disability [6–8] and acknowledges the person (patient or client) as a whole with respect for his subjective experiences and socio-cultural environment [9, 10]. In addition, it is known that Bio-Psycho-Social care should is best delivered by a multi-disciplinary approach [11]. This development is also apparent in dementia care since the cognizance that a purely biomedical model does not serve and fulfil the contemporary needs of people with dementia (PwD) and their informal caregivers.

Dementia affects 46,8 million persons worldwide and causes great stress to medical, social and informal care [12], impacting the Quality of Life of the PwD [13] but also that of its environment [14, 15]. A wide variety of programs have been developed and tested, to begin with programs mainly focusing on alleviating the cognitive impairment of the PwD by cognitive training or stimulation [16–19], all or not combined with a physical component [20]. More recently, programs also focus on reducing caregiver’s burden through e.g. psycho-education programs and cognitive behavioral therapy [21–23]. Gradually, the recognition raised that dementia not only affects cognition but also everyday functioning and autonomy in activities of daily living meaning that everything PwD do from the morning to the evening (washing, dressing, eating, cooking, doing groceries, hobbies, etc.) become hampered in a typical hierarchical way [24]. A study in community dwelling PwD showed that 70% even stopped performing certain daily activities [25]. In residential care, 51,30% of PwD didn’t have *‘anything to do’* and this number even raised to 58,7% in one year [26]. Most of them spend their day inactive [27]; though the most—and frequently unmet—needs for PwD seemed daytime activities [28]. Nonetheless their functional loss, PwD are striving to remain autonomous [25]. They want to stay involved, want to participate in decisions about their treatment, care and daily needs [29, 30], want to be *‘in control* and *feel valued’* [31]. If programs are addressing meaningful activities, autonomy and social participation positive outcomes were observed [17, 32–34]. Consequently, healthcare providers should strive to enable PwD in performing meaningful everyday activities and participation in important life roles by implementing a goal oriented, multidisciplinary and client-centered approach.

However, it is known that this new developments puts high stress on professional caregivers who already are suffering from moral distress in the health care sector [35]. Professionals ought to be able to work with the PwD as well as with the informal caregiver, approach the PwD ‘person-centered’ while considering ‘inclusion’, ‘shared decision making’, ‘coaching’, and at the same time empowering the client towards ‘self-management’, addressing meaningful daily activities and special communication skills to get connected to the PwD [36]. This requires a broad set of competencies, which are not always present in the single healthcare worker [37]. Together with the overload of administration, too little time and a lack of staff, this leaves the health care worker with a frustrating feeling of ‘willing to do the right thing but not been able to do so’ [38–40].

In order to enhance professionals’ effectiveness in reasoning and acting according to the bio-psycho-social model, the first step should be to analyze the current state of their daily practice. In a previous study published in PLOS One, we developed a self-rated scale for the professionals to evaluate their level of bio-psycho-social practice which showed good psychometric properties [41]. The final version of the questionnaire comprised 5 subscales and evaluated whether the healthcare workers were (1) working interdisciplinary and exchange information in and outside the organization (we called this first subscale networking), (2) using the client’s expertise, considering him as the central point around which the therapy/care-plan evolves (subscale 2: using clients expertise), (3) able to explore the patients’ goals, to assess all aspects of human functioning and report accordingly (subscale 3: assessment and reporting), (4) having the necessary knowledge of guidelines, tools and skills to communicate (subscale 4: professional knowledge and skills) and finally (5) involving the context of the client (subscale 5: using the environment).

The aim of this study was firstly to report on the bio-psycho-social degree in dementia care, secondly to explore its influencing factors such as setting, age, experience, professional background and job responsibilities of the professional. Alongside, it was the aim of this study to confirm the structure of the scale by means of confirmatory factor analysis and checking the internal consistency.

## Materials and methods

### Study design

This study protocol is based on the STROBE statement [42], developed to strengthen the reporting of observational studies in epidemiology. In a cross-sectional observational study design, healthcare workers in dementia care—both residential and community care—were questioned through an adapted Bio-Psycho-Social scale for use in dementia care (S1 File). The scale was supplemented with demographic and work related characteristics, considered as influencing factors for bio-psycho-social care.

### Setting and participants

This cross-sectional observational study was carried out in Flanders, Belgium’s Dutch speaking part. The Bio-Psycho-Social-Dementia-Care scale was sent out as an online survey to all organizations in dementia care, united under a Flemish umbrella organization, covering different settings in residential and community care. They were asked to pass the survey to their co-workers and thus providing a broad sample. In the sample, disciplines were stratified according to their specific focus on cure and care, therapy or social support. (a) medical doctors, nurses (with different educational levels) and other professional caregivers such as nurse assistants were stratified under the ‘Cure and Care’ group, (b) occupational-, physio-, speech & language therapists and psychologists were stratified under the ‘Therapy’ group and (c) the social, cultural, pedagogical- and family-workers under the ‘Social Support’ group. Data were collected from January till June 2016. We sought at least 310 participants sufficient for a Confirmatory Factor Analysis following the ‘subject-to-variable rule’ of 1/10 in which there are at least 10 cases for each item in the scale [43].

### The Bio-Psycho-Social Dementia Care scale

The original Bio-Psycho-Social questionnaire was developed in 3 phases: a qualitative study to determine content and face validity of the scale [44], an Exploratory Factor Analysis and test-retest reliability check [41].

The scale comprises 5 different subscales (networking, using clients expertise, assessment and reporting, professional knowledge and skills, using the environment) with a total of 31 items that have been described as statements. For instance ‘*I discussed the clinical decisions with my colleagues*’ as the first item of subscale 1 ‘*Networking’*. Each of the statements is scored by means of a Likert-scale ranging from 1 (I totally disagree with the statement) to 5 (I totally agree with the statement). Subscale-scores are calculated by summing the items divided by the number of items in the subscale. The higher the score, the better the result. The scale is constructed in a way that all statements were related to the ‘last person that was treated, coached, guided or cared for’ and the statements are formulated in the ‘I’-form. The scale shows a good to strong homogeneity (item-total ranged from 0.59–0.79) a strong internal consistency (Cronbach’s α from 0.75–0.82) and a strong test-retest reliability (Intra-Class-Correlation-Coefficient, ranging from 0.82–0.93).

For use in this study, we adapted to Bio-Psycho-Social questionnaire to be used in dementia care by replacing the word ‘client’ by PwD. This was the only change in the scale. Additional information was collected concerning demographic variables of the healthcare staff (gender, age, discipline, professional background, level of experience, clinical setting), the situation wherein the staff interacted with the PwD and the content of their work.

### Confirming the factorial structure of the adapted scale and its internal consistency

A confirmatory factor analysis was conducted to confirm the factor structure of the scale and if necessary to reduce the number of items. Maximum likelihood was used as the extraction method, and in order to maximize factor simplicity oblique rotation (promin) was used as a rotation method. To check whether the data were appropriate to conduct the confirmatory factor analysis, the Kaiser-Meyer-Olkin measure of sampling adequacy was performed, defined beforehand to be greater than 0.70 [45]. Additionally, Bartlett’s test of sphericity was performed, defined beforehand to be significant at the level of p<0.01 to ensure that the correlations that appeared in the dataset were appropriate for factor analysis. In order to determine whether the sample size was adequate to yield distinct and reliable factors, we followed the subject-to-variable rule of 10 in which there are at least 10 cases for each item in the scale [43, 46]. Items not loading strong on a factor (<0.50) were considered to delete from the final scale.

The internal consistency was expressed with a Cronbach’s alpha coefficient. The homogeneity is considered to be good if Cronbach’s alpha ranges between 0.70 and 0.95. Cronbach’s alpha was calculated for all the subscales. Items not contributing to the internal consistency (lowering the Chronbach’s Alpha) were excluded from the final subscale.

### Descriptives and differences between groups

A descriptive analysis was performed to report the healthcare professionals’ Bio-Psycho-Social competences. For every item and subscale the participants’ mean scores were calculated. Since a normal distribution of the data was observed (Shapiro-Wilk test) differences between subgroups were investigated through a student’s t-test (for 2 groups) or a one-way ANOVA with a post-Hoc Bonferroni test (for 3 groups). Differences were calculated between (a) residential community care, (b) the ‘Cure and Care’ group the Therapists group and the Social Support group, (c) juniors (< 10 year experience) seniors (> 10 years of experience). Finally, we calculated correlations (Pearson’s correlation) between the Bio-Psycho-Social score of the professionals on the one hand and the amount of time spent in practice and the years of experience of the professionals on the other hand. All statistics were performed with SPSS 24. Significance level was set a priory at 0.05.

For this study, we analyzed only the complete cases, so we didn’t have to address any missing values.

### Ethical considerations

By completing the survey all participants gave their consent to use the data for analysis.

## Results

### Study sample

Table 1 shows the characteristics of the participants. Four hundred thirteen healthcare professionals completed the questionnaire (57 men and 356 women), with a mean age of 40,74 years. The sample was heterogeneous regarding professional discipline and equally divided over residential care and community care.

**Table 1:** Characteristics of the participants (n = 413).

Age: mean (SD, range)	40,74 (11,12; 18–71)
Gender, n M/W	57/356
Years of experience in dementia care mean (sd; range)	12,70 (9,09; 0–40)
Discipline groups* (n)	
1 Cure and Care Group	160
Nurse (including head nurse)	126
Nurse assistant	55
Medical doctor	6
2 Therapists group	104
Physiotherapist	20
Occupational therapist	71
Speech and language therapist	6
Psychologist	10
3 Social Support group	100
Social worker	99
Cultural worker	20
Pedagogical worker	5
Family care	6
Extra tasks next to their initial health care discipline (n)	
Activity coach	18
Expert dementia care	58
Case manager	21
Setting (n, %)	
Community care	221 (53,50)
Residential care	192 (46,50)
Time spent with the last PwD (minutes) (n, range)	27.9 (1–120)

* Note: n is more than 100% since double disciplines occurred within the discipline groups.

### Factorial structure

The Kaiser-Meyer-Olkin measure of adequacy was 0.84, and Bartlett’s test of sphericity was statistically significant (x^2^ = 856.2, df = 465, p<0.01), meaning that the data were appropriate to conduct an exploratory factor analysis. The factor analysis was performed with a predefined and restricted number of 5 factors as defined in an exploratory factor analysis in another sample (41). The first factor, ‘networking’ has 7 items and accounted for 13,15% of the total variance. The second factor, ‘using the expertise of the client’ has 5 items and accounted for 10.38% of the total variance. The third factor, ‘assessment and reporting’, had 6 items and accounted for 10.73% of the total variance. The fourth factor, ‘professional knowledge and skills’, has 7 items and accounts for 10,14% of the total variance. Finally, the fifth factor, ‘using the environment’, has 7 items and accounted for 6.30% of the total variance. With this factor solution 50,29% of the total variance could be explained. Two items were considered to leave out of the final scale. Item 3 ‘Non-healthcare related professionals also had an important role in goal-setting for the PwD’ loaded weak (0.36) under the factor ‘networking’ and item 32 ‘My management endorses me to visit and treat the PwD in his familiar home-environment’ loaded also weak (0.36) under the factor ‘using the environment’ (See Table 2).

**Table 2:** Confirmatory factor analysis: rotated loading matrix* (n = 413).

		Factor 1	Factor 2	Factor 3	Factor 4	Factor 5
		13,15%	10.38%	10.31%	10.14%	6.30%
1	I discussed the (clinical) decisions with my colleagues	**0.74**	-	-	-	-
2	I discussed the (clinical) decision with relevant stakeholders outside my organization	**0.60**	-	-	0.39	-
3	Non-healthcare related professionals also had an important role in goal-setting for the PwD**	**0.36**	-	-	-	-
4	Healthcare professionals help each other in complex care needs.	**0.73**	-	-	-	-
5	The inter-professional cooperation in my team is good.	**0.73**	-		-	-
6	My superior is supportive when difficult decisions need to be taken.	**0.67**	-	-	-	-
7	I used the findings from my colleagues from other disciplines when listing the PwDs problems.	**0.53**	-	-	0.38	-
8	The PwD was invited to the team meetings.	0.42	**0.70**	-	-	-
9	I used the lived experience in activities of daily living of the PwD in (clinical) decision making.	-	**0.69**	-	-	-
10	I have informed the PwD about the (clinical) choices that were made.	-	**0.63**	-	-	0.36
11	My management offers me tools to enable a client-centred practice.	-	**0.69**	-		
12	The management in my unit is focused on formulating goals together with the PwD (shared goal-setting).	0.42	**0.64**	-	-	0.36
13	In our organization the PwD is always the central point around which the therapy/care-plan evolves.	-	**0.67**	-	-	-
14	I have co-created the therapy/care goals with the PwD and/or his proxies.	-	**0.63**	0.42	-	0.40
15	I used assessment tools to monitor the PwD’s wishes.	-	0.58	**0.82**	-	-
16	I used assessment tools to monitor all levels of human functioning.	-	-	**0.82**	0.32	-
17	I have access to assessment tools to assess what the PwD finds important.	-	-	**0.86**	-	-
18	In my organization we use a format of reporting that covers all aspects of human functioning.	0.446	-	**0.82**	-	-
19	I used my professional knowledge in my (clinical) decision making.	-	-	-	**0.82**	-
20	I used guidelines in (clinical) decision making.	-	-	-	**0.51**	-
21	I used my own professional experience in (clinical) decision making.	-	-	-	**0.76**	-
22	I have knowledge of different tools to assess what is important to the PwD.	-	-	0.51	**0.70**	-
23	I have the skills to approach the PwD from a holistic point of view.	-		0.54	**0.67**	-
24	I have the skills to involve the family into the therapy/care process.	-			**0.67**	0.42
25	I have the skills to defend the PwD’s choices in a team meeting		-	0.50	**0.73**	-
26	When formulating goals for the PwD, I considered the meaning of his family.	-	-	-	-	**0.71**
27	We invited the PwD (and his family) to discuss the therapy/care plan.	-	-	-	-	**0.61**
28	I worked in close collaboration with the PwD’s proxies.	-	-	-	-	**0.69**
29	I have treated the PwD at home.	-	-	-	-	**0.56**
30	I have used information about the familiar home-environment to make (clinical) decisions.	0.34	-	-	-	**0.65**
31	My management endorses me to visit and treat the PwD in his familiar home-environment**.	-	-	-	-	0.36

Labels of the factors: factor 1: networking; factor 2: using the expertise of the client; factor 3: assessment and reporting, factor 4: professional knowledge and skills, factor 5: using the environment.

* Scores beneath absolute 0.30 omitted to increase the readability.

** Items loading under 0.50 (n = 2) on one of the five factors were considered to leave out of the final scale.

### Internal consistency

The homogeneity of all subscales of the Bio-Psycho-Social Dementia Care questionnaire was fair to strong with high Cronbach’s α ranging from 0.61 to 0.78. Removing the 2 items that were considered to leave out of the scale because of a weak loading in the factor analysis did not result in significantly higher Cronbach’s α.

### Descriptive results and differences between groups

#### Differences between professionals in Residential Care and Community Care

With regard to the Residential and Community Care, we observed no significant differences for subscales 1 *‘networking’* (*t* = 0,705; p = 0,481)) and subscale 2 *‘using the expertise of the PwD’* (*t* = -0,350; p = 0,727). However, on item-level 3 items differed significantly in each of these two subscales. For the other 3 subscales, significant differences were detected. The Residential Care professionals showed a significant higher mean score for the subscale 3 *‘assessment and reporting’* (*t* = 2,309; p<0,05) and the subscale 4 *‘professional knowledge and skills’* (*t* = 3,154; p<0.01). The Community Care professionals showed a significant higher mean score for the subscale 5 *‘using the environment’* (*t* = -3,464; p<0.001). The detailed results on item level can be seen in Table 3.

**Table 3:** Comparison of the means scores of (1) the Residential Care group (RC) versus the Community Care group (CC) and (2) the juniors versus the seniors.

	Mean scores (Standard Deviation)				
	total n = 413	RC group n = 192	CC group n = 221	Juniors n = 213	Seniors n = 200
**Subscale 1: Networking**	3.74 (0.51)	3.76 (0.46	3.73 (0.59)	3,73 (0,49)	3,76 (0,54)
I discussed the clinical decisions with my colleagues.	3.82 (0.89)	3.99 (0.75)	3.68 (0.94)***	3,81 (0,8)	3,83 (0,9)
I discussed the clinical decision with relevant stakeholders outside my organization.	3.06 (1.10)	2.75 (0.66)	3.32 (1.07)***	3,04 (1,08)	3,08 (1,14)
Non-healthcare related professionals also had an important role in goal-setting for the PwD.	3.39 (0.98)	3.46 (1.02)	3.33 (0.94)	3,34 (0,96)	3,44 (1,01)
Healthcare professionals help each other with patients with in complex care needs.	4.11 (0.69)	4.12 (0.62)	4.11 (0.75)	4,11 (0,7)	4,11 (0,69)
The inter-professional cooperation in my team is good.	4.04 (0.73)	4.07 (0.70)	4.02 (0.75)	4,09 (0,74)	3,99 (0,71)
My superior is supportive when difficult decisions need to be taken.	3.93 (0.72)	4.00 (0.76)	3.86 (0.80)	4,03 (0,72)	3,82 (0,85)**
I used the findings from my colleagues from other disciplines when listing the PwD’s problems.	3.69 (0.89)	3.90 (0.75)	3.51 (0.97)***	3,63 (0,9)	3,75 (0,9)
**Subscale 2: Using the expertise of the client**	3.47(0.53)	3.46(0.0.52)	3.48 (0.53)	3,47 (0,52)	3,47 (0,54)
I used the lived experience in activities of daily living of the PwD in clinical decision making.	4.12 (0.61)	4.15 (0.61)	4.10 (0.61)	4,08 (0,63)	4,18 (0,6)
I have informed my client about the clinical choices that were made.	3.46 (0.90)	3.56 (0.85)	3.38 (0.94)*	3,46 (0,89)	3,46 (0,92)
The client was invited to the team meetings.	2.70 (1.10)	2.61 (1.09)	2.78 (1.11)	2,76 (1,14)	2,63 (1,07)
My management offers me tools to enable a client-centered practice.	3.72 (0.82)	3.70 (0.87)	3.74 (0.78)	3,76 (0,77)	3,69 (0,88)
The management in my unit is focused on formulating goals together with the PwD (shared goal-setting)	3.38 (0.93)	3.23 (0.96)	3.50 (0.89)**	3,39 (0,95)	3,36 (0,92)
In our organization the PwD is always the central point around which the therapy-plan evolves.	3.97 (0.81)	4.09 (0.80)	3.87 (0.79)**	3,94 (0,7 7)	4,01 (0,85)
I have co-created the therapy goals with the PwD and/or his proxies.	2.95 (0.94)	2.89 (0.97)	3.00 (0.93)	2,95 (0,93)	2,95 (0,97)
**Subscale 3: Assessment and reporting**	2.85 (0.74)	2.96 (0.72)	2.78 (0.74)*	3,10 (0,70)	3,13 (0,77)
I used assessment tools to monitor the PwD’s wishes.	2.83 (1.03)	2.94 (1.06)	2.73 (1.00)**	2,83 (1,01)	2,83 (1,07)
I used assessment tools to monitor all levels of human functioning	2.92 (1.03)	3.01 (1.04)	2.84 (1.03)	2,84 (0,99)	3 (1,09)
I have access to assessment tools to assess what the client finds important.	2.94 (1.03)	3.04 (1.03)	2.86 (1.03)	2,98 (1,01)	2,9 (1,07)
In my organization we use a format of reporting that covers all aspects of human functioning.	2.81 (0.85)	2.86 (0.78)	2.76 (0.89)	3,8 (0,84)	3,81 (0,85)
**Subscale 4: Professional knowledge and skills**	3.85 (0.49)	3.93 (0.46)	3.78 (0.51)**	3,82 (0,47)	3,86 (0,51)*
I used my professional knowledge in clinical decision making.	4.17 (0.57)	4.24 (0.61)	4.11 (0.63)*	4,25 (0,59)	4,01 (0,55)**
I used guidelines in my clinical decision making.	3.54 (0.90)	3.64 (0.88)	3.45 (0.91)**	3,56 (0,84)	3,53 (0,96)
I used my own professional experience in clinical decision making.	4.10 (0.68)	4.22 (0.64)	4.00 (0.71)**	3,97 (0,73)	4,24 (0,61)***
I have knowledge of different tools to assess what is important to the PwD.	3.22 (0.95)	3.29 (0.96)	3.16 (0.94)	3,25 (0,93)	3,19 (0,97)
I have the skills to approach the PwD from a holistic point of view.	4.01 (0.70)	4.19 (0.62)	3.86 (0.72)***	3,98 (0,69)	4,05 (0,71)
I have the skills to involve the family in the therapy process.	3.93 (0.68)	3.94 (0.63)	3.93 (0.73)	3,86 (0,63)	4,01 (0,73)**
I have the skills to defend the PwD’s choices in a team meeting.	3.98 (0.65)	4.03 (0.62)	3.94 (0.67)	3,92 (0,61)	4,04 (0,68)
**Subscale 5: Using the environment**	3.64 (0.60)	3.53 (0.55)	3.73 (0.62)***	3,64 (0,55)	3,63 (0,63)
We invited the PwD (and his family) to discuss the therapy plan.	3.89 (0.87)	3.93 (0.80)	3.86 (0.93)	3,93 (0,8)	3,86 (0,95)
I worked in close collaboration with the PwD’s proxies	3.67 (0.84)	3.66 (0.85)	3.85 (0.83)**	3,74 (0,85)	3,79 (0,84)
I used the family’s contribution in clinical decision making.	3.73 (0.86)	3.60 (0.83)	3.84 (0.86)**	3,7 (0,82)	3,77 (0,89)
I have used information about the familiar home-environment to make clinical decisions.	3.80 (0.78)	3.76 (0.82)	3.84 (0.74)	3,77 (0,78)	3,84 (0,79)
I have met the PwD in his familiar home-environment.	3.31 (1.22)	3.35 (1.15)	3.27 (1.29)	3,36 (1,16)	3,25 (1,3)
My management endorses me to visit and treat the PwD in his familiar home-environment.	3.34 (1.15)	2.89 (1.17)	3.72 (0.98)***	3,37 (1,13)	3,31 (1,18)

RC: Residential Care; CC: Community Care;

Students t-test:

*significant at the 0.05 level,

** significant at the 0.01 level,

*** significant at the 0.001 level.

#### Differences between junior and senior professionals

With regard to the junior and the senior professionals, a significant correlation between the years of experience and the subscale 4 *‘professional knowledge and skills’* (*r* = 0.42, p<0.05) was observed. Subsequently, we compared the junior group (less than 10 years of experience) versus the seniors and observed only significant differences on subscale 4 *‘professional knowledge and skills’* (*t* = -2.36, p<0.01). As can be seen in Table 2, the analysis on item level in subscale 4 indicated significant higher scores for the juniors on the item *‘I used my professional knowledge in clinical decision making’* in comparison with the seniors (*t* = -3.33, p<0.01). On the other hand, the seniors had significant higher values on the item *‘I used my own professional experience in clinical decision making’* in comparison with the junior healthcare professional (*t* = 4.04, p<0.01). Additionally, the seniors showed also significant higher result on the item *‘I have the skills to involve the family in the therapy process’* (*t* = 3.44, p<0.01). An additional significant difference was observed on item level for ‘*my superior is supportive when difficult decisions need to be taken’* in favor for the juniors (4,03 versus 3,82; p<0,01).

#### Differences between cure and care group, the therapy group and the social support group

In the sample, disciplines were stratified according to their specific focus on either cure and care, therapy or social support. Results are shown in Table 4. No significant differences were found in subscale 1 ‘networking’ (F = 2.77, p 0.64) and subscale 2 ‘using the expertise of the PwD’ (F = 1.07, p 0.34). Significant differences however were observed between the three groups for subscale 3 ‘assessment and reporting’ (F = 12.01, p <0.001), subscale 4 ‘professional knowledge and reporting’ (F = 19.848, p <0.000) and subscale 5 ‘using the environment’ (F = 32.741, p<0.001). Post-hoc Bonferroni tests indicated that (1) the Cure and Care group and the Therapy group had significant higher values in the subscale 3 ‘assessment and reporting’ compared to the Social Support group (respectively p 0,001 and p<0.001), (2) the Therapy group had significant higher values in subscale 4 ‘using professional knowledge and skills’ compared to the Social Support group (p 0.021) and the Cure and Care group (p<0,001), (3) the Social Support group had significant higher values in subscale 5 ‘using the environment’ compared to the Therapy group (p<0.001) and the Cure and Care-group (p<0.001).

**Table 4:** Comparison of the means scores for the cure and are group versus the therapy group and the social support group.

	Mean scores (Standard Deviation)		
	C&C group n = 160	T group n = 104	SS group n = 100
**Subscale 1: Networking**	3,73 (0,46)	3,83 (0,50)	3,66 (0,62)
I discussed the clinical decisions with my colleagues.	3,91 (0,69)	4,09 (0,66)	3,32 (1,07)
I discussed the clinical decision with relevant stakeholders outside my organization.	2,86 (1,04)	2,89 (1,17)	3,57 (1,01)
Non-healthcare related professionals also had an important role in goal-setting for the PwD.	3,5 (0,92)	3,28 (1,09)	3,21 (0,97)
Healthcare professionals help each other with patients with in complex care needs.	4,11 (0,67)	4,13 (0,64)	4,13 (0,79)
The inter-professional cooperation in my team is good.	4,11 (0,65)	4,12 (0,76)	3,98 (0,78)
My superior is supportive when difficult decisions need to be taken.	4,01 (0,66)	3,9 (0,91)	3,92 (0,85)
I used the findings from my colleagues from other disciplines when listing the PwD’s problems.	3,69 (0,75)	3,92 (0,9)	3,32 (1,05)^b^**^,c^***
**Subscale 2: Using the expertise of the client**	3,43 (0,52)	3,49 (0,55)	3,52 (0,53)
I used the lived experience in activities of daily living of the PwD in clinical decision making.	4,1 (0,55)	4,25 (0,57)	4,07 (0,57)
I have informed my client about the clinical choices that were made.	3,44 (0,87)	3,64 (0,9)	3,31 (0,93)
The client was invited to the team meetings.	2,53 (1,05)	2,61 (1,1)	3 (1,13) ^b^**
My management offers me tools to enable a client-centered practice.	3,72 (0,79)	3,69 (0,87)	3,8 (0,78)
The management in my unit is focused on formulating goals together with the PwD (shared goal-setting)	3,34 (0,86)	3,21 (1)	3,63 (0,92) ^c^**
In our organization the PwD is always the central point around which the therapy-plan evolves.	3,97 (0,76)	4,05 (0,87)	3,82 (0,8)
I have co-created the therapy goals with the PwD and/or his proxies.	2,91 (0,92)	2,97 (1,04)	3,06 (0,85)
**Subscale 3: Assessment and reporting**	3,26 (0,66)	3,19 (0,78)^a^***	2,82 (0,73)^b^***
I used assessment tools to monitor the PwD’s wishes.	3,01 (0,96)	2,83 (1,16)	2,57 (0,91)^b^**
I used assessment tools to monitor all levels of human functioning	3,08 (0,94)	3,1 (1,13)	2,56 (0,97)^b^***^,c^***
I have access to assessment tools to assess what the client finds important.	3,03 (1)	3,11 (1,06)	2,62 (0,99)^b^**^,c^**
In my organization we use a format of reporting that covers all aspects of human functioning.	3,94 (0,72)	3,77 (0,85)	3,55 (1,03)^b^**
**Subscale 4: Professional knowledge and skills**	3,88 (0,45)	4,03 (0,43)^a^***	3,63 (0,51)^c^***
I used my professional knowledge in clinical decision making.	4,16 (0,54)	4,35 (0,52)^a^*	4,04 (0,51) ^c^***
I used guidelines in my clinical decision making.	3,61 (0,86)	3,66 (0,92)	3,26 (0,87)^b^**^,c^**
I used my own professional experience in clinical decision making.	4,12 (0,57)	4,25 (0,71)	3,91 (0,75) ^c^***
I have knowledge of different tools to assess what is important to the PwD.	3,37 (0,91)	3,32 (0,9)	2,95 (1,00)^b^**^,c^*
I have the skills to approach the PwD from a holistic point of view.	4,02 (0,63)^a^***	4,34 (0,6)	3,64 (0,72) ^b^***^,c^***
I have the skills to involve the family in the therapy process.	3,91 (0,59)	4,07 (0,66)	3,81 (0,8) ^c^**
I have the skills to defend the PwD’s choices in a team meeting.	3,94 (0,63)	4,19 (0,48)^a^**	3,8 (0,73) ^c^***
**Subscale 5: Using the environment**	3,47 (0,57)	3,53 (0,59)	4,01 (0,46)^b^***^,c^***
We invited the PwD (and his family) to discuss the therapy plan.	3,75 (0,93)	3,92 (0,88)	4,04 (0,79) ^b^*
I worked in close collaboration with the PwD’s proxies	3,58 (0,84)	3,80 (0,87)	4,05 (0,74) ^c^***
I used the family’s contribution in clinical decision making.	3,53 (0,88)	3,62 (0,84)	4,12 (0,69)^b^***^,c^***
I have used information about the familiar home-environment to make clinical decisions.	3,66 (0,85)	3,91 (0,69)	3,9 (0,66)
I have met the PwD in his familiar home-environment.	3,14 (1,22)	3,01 (1,38)	3,96 (0,76)^b^***^,c^***
My management endorses me to visit and treat the PwD in his familiar home-environment.	3,16 (1,04)	2,92 (1,24)	4,04 (0,85) ^b^***^,c^***

C&C: Cure and Care group; T: Therapy group; SS: Social Support Group;

Post Hoc Bonferonni: a: differences between C&C and T group; b: differences between C&C group and the SS group; c: differences between the T group and the SS group.

*significant at the 0.05 level,

** significant at the 0.01 level,

*** significant at the 0.001 level.

#### The relation between the time spent with the PwD and the level of Bio-Psycho-Social competence of the professional

The mean time spent on the last session with the PwD was 27.9 minutes (SD 23.44), with a range from 1 minute to 120 minutes. There is a small, but significant correlation between the amount of time and the subscale 2 *‘using the expertise of the PwD’* (r = 0.28, p<0.01), and the subscale 5 *‘using the environment’* (r = 0.34, p<0.01), meaning that the more time spent during the session, the more the own capability of the PwD was used and the more was worked together with and within the environment of the PwD.

## Discussion

Healthcare professionals in dementia care are expected to work according to a Bio-Psycho-Social perspective [38]. To date, the focus on biomedical aspects and task completion still seems to dominate [37], presenting a gap between the rhetoric and the reality of working within a Bio-Psycho-Social model. The professional is solicited to use a broad set of competencies which are not always present in the single healthcare worker. Therefore, we aimed to evaluate the Bio-Psycho-Social approach in dementia care and set out to adapt the original Bio-Psycho-Social questionnaire towards dementia care. The Confirmatory Factor Analysis confirmed on the one hand the theory of Bio-Psycho-Social practice in which (a) putting the client central, (b) working in close collaboration with the context of the individual and (c) a good communication between all disciplines is advocated [9] and on the other hand the results of a preceding qualitative study on which the item derivation of the questionnaire was based [44]. At the same time the factor structure of the original Bio-Psycho-Social Scale has been confirmed. However, within the Factor Analysis, 2 items didn’t load strong. We considered whether these items should be removed from the final scale, but we decided not do so because of following reasons: (a) the strong internal consistency, (b) the rationale that these items were considered as important by an expert panel. Additionally, we could have performed a Confirmatory Factor Analysis for the different groups within the sample, but we considered the groups too small to draw valid conclusions. In addition, other remarks need to be made concerning the sample. The digital survey has been sent out to an unknown sample of healthcare workers through the umbrella organizations. Consequence of this technique of data gathering is that we do not known if the sample is representative for the entire population. However, the results showed that the sample included a variety of professionals from various settings and different ages which offered opportunities for further exploration. By running the scale in a sample with different settings and professionals with a different background and experience, we discovered that the Bio-Psycho-Social-Dementia-Care scale was able to detect differences between healthcare professionals in their BPS-practice, which adds to the clinical usefulness for this scale and creates opportunities for organizations to launch improvement projects, which are of paramount importance in quality improvement and assurance in healthcare delivery [47]. However, at this point, it is not known whether the instrument will be able to detect changes over time, which is absolutely necessary since the goal of this instrument is to improve healthcare delivery. Consequently, further research on the responsiveness is desirable. However, we considered the questionnaire psychometrically sound, delivering valid and reliable results worth to discuss.

Globally healthcare professionals in dementia care are doing fine. Of course, one can argue that a weakness of the study is that the professionals are asked to evaluate themselves. It is indeed generally accepted that respondents tend to report socially desirable answers and that external report (e.g. PwD and their informal caregivers) might be more objective. However, besides the fact that we doubt that patients and proxies are more objective, it is also known that considering one’s own professional functioning might lead to better awareness of what can be considered as good care. Moreover, by reflecting on an actual situation in which they were engaged with the PwD, the participants felt personal addressed which might have led to reliable answers. Nevertheless, completing this questionnaire with a client and proxy-report version might be a good idea.

Despite the risk for socially desirable answers, the lowest performance is seen in the subscale ‘assessment and reporting’ by either professionals in Residential Care as in Community Care. The results show that both groups don’t have sufficient access to assessment instruments and don’t use them accordingly. Assessment however is described as a prerequisite for accurate goal-setting [48–52]. Given the tendency in contemporary healthcare to evolve from a problem-oriented towards a goal-oriented care [48], it is still a huge step to be taken to assess and evaluate goals and wishes of the PwD [38]. A solid and feasible system for evaluating and reporting, preferably on a digital platform that cut across the different settings, may be needed to improve the quality of care and the efficiency of Bio-Psycho-Social practice [49]. Results indicate that Residential Care professionals use more assessment instruments compared to Community Care. Probably the Residential Care is more focused on the diagnostic phase of the clinical reasoning process because it is mandatory while Community Care tends to be more focused on the actual care phase [50]. But either Residential or Community Care, assessment and reporting are considered of utmost importance in Bio-Psycho-Social practice for different reasons. An accurate assessment [and accordingly reporting] is necessary to identify all relevant factors to identify goals and it helps in liaising with all other team-members, with other teams and with other organizations [51]. Certainly when boundaries between disciplines tend to fade away, the insidious risks may exist that information will not be gathered, or will be double gathered or will get lost.

Secondly, the subscale ‘using the environment’ showed room for improvement. In this study, the Community Care professionals seemed here to be more competent. This seems not surprising since they primarily work with and even in the environment of the PwD contrary to professionals in Residential Care. However, the latter do work constantly ‘in’ the environment of the PwD, but maybe they consider the nursing home more as ‘their working environment’ than as ‘home environment’ of the PwD’s [37]. Anyway, a deliberate involvement of the entourage has a positive effect on the effectiveness of a therapy program in Community Care [53].

Thirdly, the subscale ‘networking’, showed decent scores in Residential and in Community Care, indicating that this aspect of Bio-Psycho-Social practice is fair to good. Networking referring to interdisciplinary working in and outside the organization [54], encompasses a continuous consultation between healthcare professionals, featured by a strong collaboration in and outside the organization during the entire trajectory of the PwD. Although the decent scores, room for improvement might be present, certainly when looking at the wide range in the scores (from 1 to 5). Interdisciplinary communication, exchange of information and written reports seem pivotal for good care.

The subscale ‘using the expertise of the PwD’ refers to the extent of the client-centered attitude of the healthcare professional and clearly indicated opportunities for action. Several studies showed that healthcare professionals doubt the capacities of PwD to make decisions since they assume that cognitive decline automatically results in hampered decision making [55, 56] However, PwD do want to take their own decisions and are capable to do so, even in more severe dementia [30, 57, 58]. It is however important do know which type of decisions the person wants to be involved in: everyday care, medical treatment or long term placement [30].

Doyle [37] described the biomedical model as ‘a default care model’ and the Bio-Psycho-Social model as more positive, looking into strengths, capacities rather than deficits. Hearing the voice of the PwD and being more engaged in communication and mutual exchange of information, helps to ‘humanize’ the PwD and supports him to achieve his goals. As Bright argued: ‘Being with’ clients might be more important than *‘doing to’* them. Consequently; shifting from *‘what can I do for this person’* to *‘who is this person and what does he need’* is strongly advised [59]. Such an attitude may be necessary to promote a person-centered approach [59]. Unfortunate, in dementia care, health care professionals are not grasping all opportunities to sustain personhood for people with dementia [60]. Person centered care however has shown significant beneficial effects on decreasing behavioral symptoms and psychotropic medication use in dementia residents in long-term care [61].

At last, the subscale ‘professional knowledge and skills’ showed the highest score of all subscales, though with a significant difference between the seniors and the juniors. This is worth considering since it is known that novices do have more knowledge than experienced professionals, but the latest have more skills (e.g. communication, emotional approaches, etc.) than novices [38]. However, both aspects (knowledge and skills to work accordingly), are considered as essential. An interesting finding in this study was (data not shown in the Results) that the knowledge from the junior meets the skills of the senior when the professional has 12 years of experience in working with PwD. At this point in the career, the healthcare professional seemed to use at his best both his expert knowledge and his experienced skills. He combines the best of both features into the optimal Bio-Psycho-Social practice. This finding highlights the strengths of both groups of professionals, the juniors can be impelled to train the seniors in gaining knowledge, and the seniors can be prompted to train the juniors in acquiring skills. However, our study also showed that novices felt more ‘supported’ by the management than seniors. This finding stresses the need to compose heterogeneous teams with regards to age and expertise but also the need of life-long learning when working with PwD. A reciprocal program of hands-on training, education and inter-professional collaboration might offer opportunities [38]. Ideally, existing basic curricula should be expanded with aspects of ‘dementia positivity’ [62]. The Bio-Psycho-Social Dementia Care questionnaire can be used in curricula to evaluate students, but also to organize ‘tailored’ education for healthcare teams.

As last remark, one can doubt if healthcare professionals should be so highly skilled to cope with the Bio-Psycho-Social demands. Certainly when already 14% of them find their work ‘not workable’, with as major risk factors workload and in particular emotional load (Flemish report on work, 2013). In residential care particularly, the amount of moral distress is extremely high, namely 35% of the professionals reported to be stressed. The Behavior, Psychological Symptoms of Dementia—with agitation as most destroying behavior—are negatively impacting job satisfaction [63]. However, increased knowledge of management of dementia could help professional caregivers to reduce their work-related stress and to improve Quality of Life of PwD [64]. Thus, continuous education of the professionals looks beneficial and not imposing and—moreover—can be seen as a human right of each PwD.

## Conclusion

The current status of the Bio-Psycho-Social practice in dementia care is globally good, though there is room for improvement and the findings of this study offer opportunities to do so. It is recommended to work interdisciplinary in and outside the organization while working in close but deliberate collaboration with the PwD and his or her environment. As messages for the management, we may advise that it is pivotal to combine the strengths of the different occupational groups within the team, since they have complementary competencies. Moreover, since we showed that knowledge and skills in dementia care are at its best after 12 years of experience, it is advisable to install a heterogeneous team with regard to age and experience. Finally, investing in easy to use assessment and reporting systems might be beneficial to improve dementia care.

## Supporting information

S1 Dataset Underlying dataset for the Bio-Psycho-Social-Dementia-Care scale. (XLSX)

S1 File The Bio-Psycho-Social-Dementia-Care scale. (DOCX)
