# Supplementary material for: Quality in dementia care: A cross sectional study on the Bio-Psycho-Social competencies of health care professionals
Source: PLoS One. 2018 Feb 1;13(2):e0191440. doi: 10.1371/journal.pone.0191440 (PMC5794079; doi:10.1371/journal.pone.0191440)
Supplement: S1 File — (DOCX) [file pone.0191440.s002.docx]

**The Bio-Psycho-Social-Dementia-Care scale.**

By answering the questions, please keep your last client in mind.

Response options for all items is each subscale: 1: I totally disagree to 5: I totally agree

PwD = Person with Dementia

**Subscale 1: Networking**

1. I discussed the clinical decisions with my colleagues.
2. I discussed the clinical decision with relevant stakeholders outside my organization.
3. Non-healthcare related professionals also had an important role in goal-setting for the PwD.
4. Healthcare professionals help each other when PwD have complex care needs.
5. The inter-professional cooperation in my team is good.
6. My superior is supportive when difficult decisions need to be taken.
7. I used the findings from my colleagues from other disciplines when listing the PwD’s problems.

**Subscale 2: Using the expertise of the PwD**

1. I used the lived experience of the PwD in clinical decision making.
2. I have informed the PwD about the clinical choices that were made.
3. The PwD was invited to the team meetings.
4. My management offers me tools to enable a client-centered practice.
5. The management in my unit is focused on formulating goals together with the PwD (shared goal-setting).
6. In our organization the PwD is always the central point around which the therapy-plan evolves.
7. I have co-created the therapy goals with the PwD and/or his proxies.

**Subscale 3: Assessment and reporting**

1. I used assessment tools to monitor the PwD’s wishes.
2. I used assessment tools to monitor all levels of human functioning.
3. I have access to assessment tools to assess what the PwD finds important.
4. In my organization we use a format of reporting that covers all aspects of human functioning.

**Subscale 4: Professional knowledge and skills**

1. I used my professional knowledge in clinical decision making.
2. I used guidelines in my clinical decision making.
3. I used my own professional experience in clinical decision making.
4. I have knowledge of different tools to assess what is important to the PwD.
5. I have the skills to approach the PwD from a holistic point of view.
6. I have the skills to involve the family into the therapy process.
7. I have the skills to defend the PwD’s choices in a team meeting.

**Subscale 5: Using the environment**

1. We invited the PwD (and his family) to discuss the therapy plan.
2. I worked in close collaboration with the PwD’s proxies.
3. I used the family’s contribution in clinical decision making.
4. I have used information about the familiar home-environment to make clinical decisions.
5. I have met the PwD in his familiar home-environment.
6. My management endorses me to visit and treat the PwD in his familiar home-environment.
